# A founder *COL4A3* pathogenic variant resulting in Alport syndrome and thin basement membrane disease: a case report series

**DOI:** 10.3389/fmed.2023.1281049

**Published:** 2023-12-21

**Authors:** Tinatin Tkemaladze, Kakha Bregvadze, Eka Kvaratskhelia, Elene Abzianidze, Tinatin Davitaia

**Affiliations:** ^1^Department of Molecular and Medical Genetics, Tbilisi State Medical University, Tbilisi, Georgia; ^2^Department of Pediatrics, Givi Zhvania Pediatric Academic Clinic, Tbilisi State Medical University, Tbilisi, Georgia; ^3^Department of Pediatric Nephrology, M. Iashvili Children's Central Hospital, Tbilisi, Georgia

**Keywords:** Alport syndrome, thin basement membrane disease, *COL4A3*, consanguinity, founder effect

## Abstract

Alport syndrome is a rare genetic condition characterized by kidney disease, hearing impairment, and ocular abnormalities. It exhibits various inheritance patterns involving pathogenic variants in *COL4A3*, *COL4A4*, and *COL4A5* genes. The phenotypes can range from isolated hematuria with a non-progressive or very slowly progressive course to progressive kidney disease with extrarenal abnormalities. Timely diagnosis of Alport syndrome facilitates the early and effective implementation of treatment, as well as genetic counseling. Here, we report the *COL4A3* c.765G > A, p.((=)) mutation in three ethnically Azerbaijani, apparently unrelated, consanguineous families from the village of Algeti in the Marneuli region of Georgia. We speculate that this variant could represent a founder mutation within this population and recommend offering genetic testing to Algeti village residents with persistent hematuria.

## Background

Alport syndrome is one of the most commonly inherited kidney diseases, with population prevalence varying from 1 in 5,000 to 1 in 53,000 (1). Inheritance of Alport syndrome can be X-linked (OMIM 301050) due to pathogenic variants in *COL4A5*, autosomal recessive (OMIM 203780) caused by biallelic pathogenic variants in *COL4A3* or *COL4A4*, autosomal dominant (OMIM 104200) caused by heterozygous *COL4A3* or *COL4A4* variants, or digenic, with variants in two of the *COL4A3*–*COL4A5* genes (2). Individuals with heterozygous pathogenic variants in *COL4A3* or *COL4A4* genes are considered carriers of autosomal recessive Alport syndrome and are often diagnosed with thin basement membrane nephropathy (TBMN; also called thin basement membrane disease). However, there is no universal consensus on the terminology and some guidelines use the term “COL4A3 and COL4A4 heterozygotes” (2, 3).

Alport syndrome is a clinically heterogenous condition (4). Typically, recurrent microscopic or gross hematuria is the first sign of Alport syndrome, and it is usually detected in childhood. Progressive nephropathy leads to end-stage kidney disease (ESKD) either in early or middle adulthood (5). Sensorineural hearing loss and non-nephrotic range proteinuria are also common findings (6). Ocular abnormalities affecting the cornea, lens, and retina may be present as well (7). The clinical features of nephropathy are usually more severe and progressive in male than female patients. Heterozygous individuals with pathogenic variants in *COL4A3* or *COL4A4* genes have TBMN and present with persistent hematuria, occasional proteinuria, hypertension, and kidney dysfunction. A systematic review conducted by Matthaiou and colleagues demonstrated that approximately 29% of heterozygous patients developed chronic kidney disease and 15.1% reached ESKD (mean age of 52.8). Hearing loss or ocular abnormalities typical of X-linked Alport syndrome have low prevalence (8). It should be noted that many women with X-linked Alport syndrome have isolated microscopic hematuria (9). Patients with digenic *COL4A3* and *COL4A4* variants have increased risk of proteinuria, and the phenotype is intermediate between autosomal dominant and autosomal recessive Alport syndrome (10).

*COL4A3–COL4A5* genes are characterized by significant allelic heterogeneity, with missense variants being the most common type of mutations, and nonsense variants resulting in nonsense-mediated mRNA decay. Rarely, large deletions and synonymous splice-site variants resulting in abnormal splicing have also been reported (1). Potential splicing effects can be examined with prediction tools such as MaxEntScan or SpliceSiteFinder, and eventually require confirmation with *in vitro* or *in vivo* functional studies or RNA sequencing.

Timely diagnosis of Alport syndrome is crucial as effective inexpensive treatment with the renin-angiotensin-aldosterone system (RAAS) blockade delays the development of kidney failure (11). However, diagnosis is often difficult on the basis of clinical features, family history, and even kidney biopsy. Genetic testing is sensitive and accurate, and provides information on the mode of inheritance.

Here, we describe a *COL4A3* c.765G > A, p.((=)) variant in the homozygous and heterozygous state in three consanguineous families from Algeti village, Marneuli region, Georgia (population of 4,253), where 99.6% of the village residents are ethnic Azerbaijani. We speculate that this variant could be a founder mutation in this population.

## Case series

### Family 1

**Figure 1:**
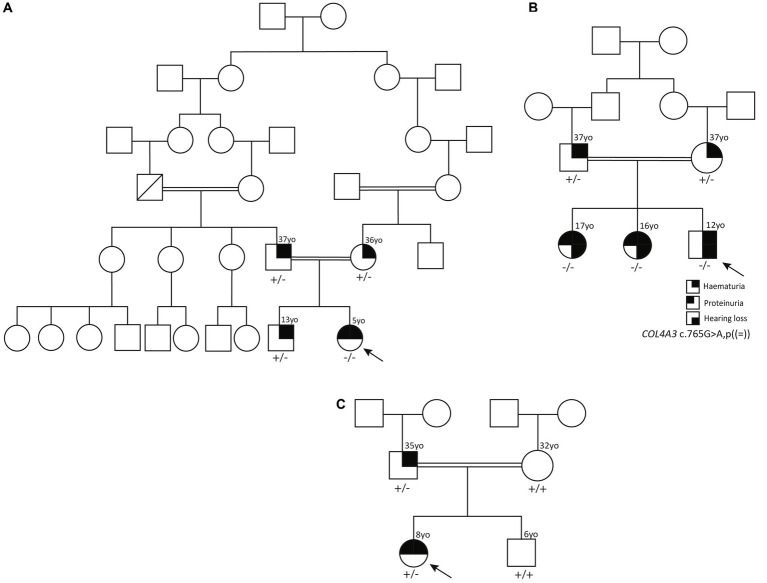
**(A)** Pedigree of family 1 showing the high degree of consanguinity. **(B)** Pedigree of family 2. **(C)** Pedigree of family 3. “+” indicates a wild type allele and “−” corresponds to the *COL4A3* c.765G > A variant.

The proband is a 5-year-old girl with asymptomatic microscopic hematuria, proteinuria, and leukocyturia detected by routine urine analysis. She was born at term as the second child of healthy, consanguineous ethnic Azerbaijani parents living in Algeti village, Marneuli region, Georgia (Figure 1A). Her developmental milestones were normal. The physical examination was unremarkable, with no edema. Her heart rate was normal (80 beats/min) and regular. She also had a normal blood pressure (90/60 mm Hg). The slit-lamp examination was normal.

Urine analysis (UA) was remarkable for protein + and erythrocytes +++. Urine microscopy revealed >1,600 red blood cells (RBC) and 8 white blood cells (WBC) per high-powered field. Phase-contrast microscopy of the urine revealed dysmorphic erythrocytes >40% and acanthocytes <5%. The spot urine protein/creatinine ratio (UPCR) was 719 mg/g (<200 mg/g). Her serum electrolyte, urea, and creatinine values were normal. Further serological tests (C_3_, C_4_, antinuclear antibodies (ANA), antineutrophilic cytoplasmic antibodies (ANCA), and circulating IgA) were within normal ranges. Abdominal and kidney ultrasound examinations were unremarkable. UA performed in both parents and her 13-year-old brother demonstrated mild hematuria (+).

Considering a positive family history of hematuria, whole exome sequencing (WES) was performed, which revealed the homozygous, likely pathogenic variant c.765G > A p.((=)) in the *COL4A3* gene (NM_000091.4). The variant segregated in the heterozygous state in the parents and the brother.

Treatment with the angiotensin-converting enzyme inhibitor (ACEI) ramipril 1.5 mg/m^2^/once daily/orally was initiated. The patient had good adherence to the medication, without any side effects. There was a reduction in hematuria, and no significant rebound proteinuria was present after 2 years of treatment. Currently, her UPCR is 324 mg/g. Her pure-tone audiometry was normal.

### Family 2

The proband is a 12-year-old boy, in whom routine UA revealed microscopic hematuria at the age of 10. He was born at term as the third child of healthy, consanguineous Azerbaijani parents from Algeti village (Figure 1B). He had age-appropriate psychomotor development. The physical examination did not show any signs of edema. His heart rate (80 beats/min) and blood pressure (90/60 mm Hg) were normal. Abdominal and kidney ultrasound were unremarkable. His first UA revealed hematuria +++ and no proteinuria. Urine microscopy was remarkable for 238 RBC per high-powered field. Phase-contrast microscopy of the urine revealed dysmorphic erythrocytes <40% and acanthocytes 18%; spot UPCR was 180 mg/g. Abdominal and kidney ultrasound examinations were unremarkable.

UA and urinary microscopy were performed in both parents and his 16-year-old and 17-year-old sisters. Both sisters and the mother had hematuria +++, whereas the father had mild hematuria +. Additionally, both sisters revealed proteinuria +++, but not the parents.

WES was performed in the proband, and the homozygous variant c.765G > A, p.((=)) in the *COL4A3* (NM_000091.4) gene was identified. The variant segregated in the heterozygous state in both parents and in the homozygous state in both sisters.

Treatment with ACEI (ramipril 1.5 mg/m^2^/once daily/orally) was initiated in all siblings. The patients had good adherence to the medication, without any side effects. After 2 years of treatment, proteinuria and hematuria persisted. Recent pure-tone audiometry revealed unilateral moderate sensorineural hearing loss in the proband and bilateral sensorineural hearing loss in both sisters.

### Family 3

The proband is an 8-year-old girl who presented with dark urine at 4 years of age. She was born from consanguineous Azerbaijani parents from Algeti village (Figure 1C). UA revealed hematuria (+++) and proteinuria (+), and urine microscopy was remarkable for 374 RBC per high-powered field. Phase-contrast microscopy of the urine revealed dysmorphic erythrocytes >40% and acanthocytes >5%. The spot UPCR was 183 mg/g. The physical examination was unremarkable and the heart rate, blood pressure, CBC, and slit-lamp examination were all normal.

UA was also performed in both parents; the father revealed hematuria +++ and the mother had normal UA. WES was performed in the proband, which detected the heterozygous variant c.765G > A, p. ((=)) in the *COL4A3* (NM_000091.4) gene. The variant segregated in the father in the heterozygous state.

Treatment with ACEI (ramipril 1.5 mg/m2/once daily/orally) was initiated. The patient had good adherence to the medication, without any side effects. Her second UA 1 month later was negative for proteinuria but positive for hematuria +++. Urine microscopy showed 30 RBC per high-powered field. Her pure-tone audiometry was normal.

## Discussion

The *COL4A5*, *COL4A3*, and *COL4A4* genes involved in Alport syndrome code for the collagen IV α5-, α3-, and α4-chains. Type IV collagen is a major structural component of the glomerular basement membrane (GBM). Mutations in α5-, α3-, and α4-chains may result in disrupted folding and assembly of monomers, which are then rapidly degraded in the cell. As a result, there is a switch, with persistence of α1:α1:α2 networks in the mature GBM. The α1:α1:α2 network is less resistant to higher intraglomerular pressure and proteolytic attacks compared to the α3:α4:α5 network (12).

Heterozygous mutations in *COL4A3/COL4A4* are associated with a spectrum of phenotypes, ranging from complete absence of detectable symptomatology through isolated, asymptomatic hematuria to progressive kidney disease, sensorineural hearing loss, and ocular abnormalities (2). Recent data from the 100,000 Genomes project provide evidence that heterozygous variants in *COL4A3/COL4A4* genes are present in approximately 1% of Europeans, which indicates that monoallelic variants may not significantly reduce fitness by producing a milder phenotype without ESKD (13). However, even within families, the phenotype associated with monoallelic mutations in *COL4A3/COL4A4* genes can vary significantly. The underlying causes for clinical heterogeneity are likely diverse, including modifier genes that disrupt the effects of mutations on the synthesis, assembly, or function (or a combination of these) of α345(IV)21 and non-genetic factors such as smoking, hypertension, and dietary habits involving the consumption of salt and animal protein. The phenotype resulting from heterozygous pathogenic variants in *COL4A3/COL4A4* is transmitted in an autosomal dominant manner, and the exact genotype–phenotype correlations have yet to be established.

Interpretation of DNA sequence variants and assessment of their pathogenicity are not always straightforward and simple, often requiring functional studies and computational tools. Generally, the interpretation of frameshift and nonsense variants poses less of a challenge compared to missense variants, whereas synonymous changes are the most challenging to interpret. As previously thought, synonymous variants are not necessarily silent, and evidence suggests that codon usage determines gene expression and splicing as well as protein function or conformation, especially if the variant is located close to the regulatory, donor, or acceptor splice site (14, 15). Moreover, several studies have shown that 15–60% of human genetic diseases are caused by splicing variants (16, 17).

The *COL4A3* synonymous variant c.765G > A described in our series of patients is predicted not to change the amino acid sequence and is found at a very low frequency in the gnomAD v2.1.1 dataset (total allele frequency: <0.001%). It is located at the last nucleotide of exon 13. *In silico* predictions from MutationTaster suggest it to be deleterious, while Human Splicing Finder, NNSPLICE, NetGene2, and MaxEntScan indicate that it may affect splicing. A recent study employed an *in vitro* minigene assay, demonstrating that the *COL4A3* variant c.765G > A affects pre-mRNA splicing by skipping exon 13 and consequently leading to an in-frame deletion of exon 13. Based on the data, the variant c.765G > A was reclassified as “pathogenic” (PVS1, PS3, PM2, PP3) according to the ACMG guidelines and the minigene assay results (18).

**Figure 2:**
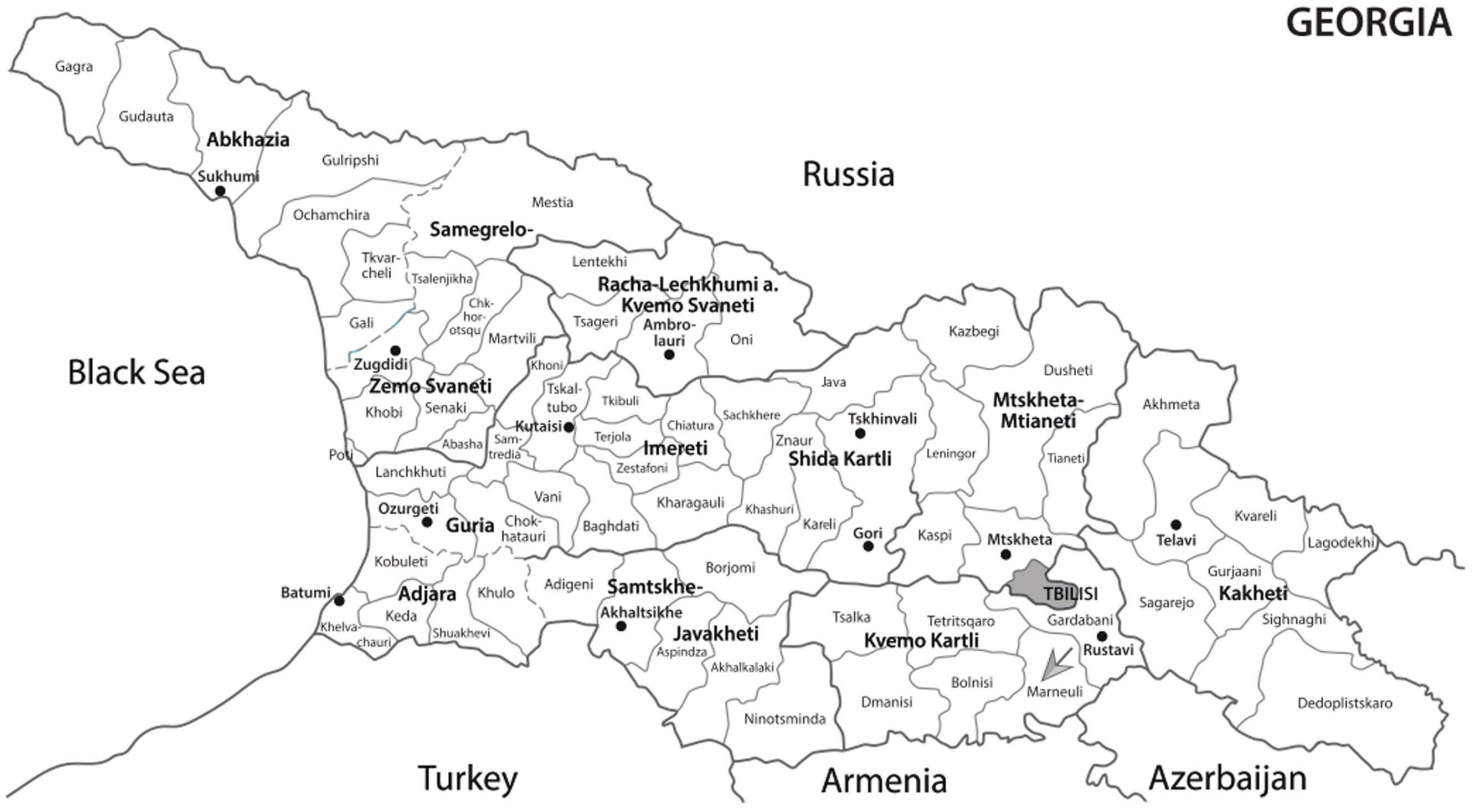
A map of Georgia with the Marneuli region indicated by the arrow.

It is noteworthy to mention that the three families described in our study are from the same small village of Algeti (population of 4,253), Marneuli region, South Georgia (Figure 2). The Marneuli region shares a border with Azerbaijan and has a population of approximately 108,000, with 83.8% of its residents being ethnic Azerbaijanis. Azerbaijanis constitute 6.5% of Georgia’s population and form the country’s largest ethnic minority, primarily residing in the rural areas of Southern Georgia. Cousin marriage is a prevalent practice among Marneuli residents. Historically, beginning in the 16th century, several villages in the southern part of Georgia were settled by Qizilbash tribes, which gradually expanded into nearby eastern and western lands throughout the 18th century (19, 20).

The identification of a *COL4A3* c.765G > A variant in carriers and homozygotes from three apparently unrelated families in the same village, along with the community’s historical background, suggest a possible founder effect. A recent demonstration of a founder effect was described in an isolated Czech Romani population, where two variants in the *COL4A4* and *COL4A3* genes contributed to a high prevalence of kidney failure (21). Currently, there is almost no literature describing genetic diseases from the Marneuli region of South Georgia, except for a single case report involving siblings with ultra-rare SC4MOL deficiency and a novel mutation in the *MSMO1* gene (22).

Studies conducted in consanguineous families from isolated populations have enabled the discovery of new disease-causing alleles and disease genes, sometimes leading to the identification of more than one genetic disease in affected individuals (23, 24). A future large-scale study of the Marneuli population could significantly contribute to advancing such discoveries. The identification of a specific mutation in several families from the same area of Algeti village in Georgia, coupled with information about the community’s history, suggest that the c.765G > A variant in the *COL4A3* gene likely results from a combination of a founder effect and consanguinity. Residents of Algeti village with persistent hematuria should be offered genetic testing to facilitate the early and effective implementation of treatment, as well as genetic counseling. Investigating the presence of founder mutations in isolated populations could potentially enhance the understanding of disease mechanisms, progression, and therapeutic strategies.

This study has several limitations. The lack of kidney biopsy data limits our ability to provide direct histological correlations with the observed clinical presentations and genetic findings. It would be valuable to conduct a large-scale study among Algeti village residents to identify individuals with hematuria and to make a detailed characterization of the phenotype and genotype to definitively establish the founder effect. Our study provides insights into the initial diagnosis and management of individuals with the *COL4A3* c.765G > A variant. However, long-term follow-up data are lacking. The natural history of the disease, including the progression of kidney dysfunction and extrarenal manifestations, remains to be determined in this population. The focus of our study was on a specific *COL4A3* c.765G > A variant. It is important to note that the phenotypic expression of Alport syndrome can vary significantly depending on the specific variant involved. Therefore, our findings may not entirely apply to individuals with different *COL4A3* variants.

## Data availability statement

The original contributions presented in the study are included in the article/supplementary material, further inquiries can be directed to the corresponding author.

## Ethics statement

The studies involving humans were approved by Tbilisi State Medical University Ethics Committee. The studies were conducted in accordance with the local legislation and institutional requirements. Written informed consent for participation in this study was provided by the participants’ legal guardians/next of kin. Written informed consent was obtained from the individual(s) for the publication of any potentially identifiable images or data included in this article. Written informed consent was obtained from the participant/patient(s) for the publication of this case report.

## Author contributions

TT: Conceptualization, Investigation, Writing – original draft, Writing – review & editing, Supervision. KB: Conceptualization, Investigation, Writing – original draft, Writing – review & editing, Methodology. EK: Visualization, Writing – review & editing. EA: Writing – review & editing. TD: Investigation, Supervision, Writing – review & editing.
